# Effective Gold Nanoparticle-Antibody-Mediated Drug Delivery for Photodynamic Therapy of Lung Cancer Stem Cells

**DOI:** 10.3390/ijms21113742

**Published:** 2020-05-26

**Authors:** Anine Crous, Heidi Abrahamse

**Affiliations:** Laser Research Centre, Faculty of Health Sciences, University of Johannesburg, P.O. Box 17011, Doornfontein, Johannesburg 2028, South Africa; aninecrous@gmail.com

**Keywords:** lung cancer stem cells, gold nanoparticles, immunoconjugate, photodynamic therapy, cytotoxicity, cell death

## Abstract

Cancer stem cells (CSCs) are a leading contributor to lung cancer mortality rates. CSCs are responsible for tumor growth and recurrence through inhibition of drug-induced cell death, decreasing the effect of traditional cancer therapy and photodynamic therapy (PDT). PDT can be improved to successfully treat lung cancer by using gold nanoparticles (AuNPs), due to their size and shape, which have been shown to facilitate drug delivery and retention, along with the targeted antibody (Ab) mediated selection of CSCs. In this study, a nanobioconjugate (NBC) was constructed, using a photosensitizer (PS) (AlPcS_4_Cl), AuNPs and Abs. The NBC was characterized, using spectroscopy techniques. Photodynamic effects of the NBC on lung CSCs was evaluated, using biochemical assays 24 h post-irradiation, in order to establish its anticancer effect. Results showed successful conjugation of the nanocomposite. Localization of the NBC was seen to be in integral organelles involved in cell homeostasis. Biochemical responses of lung CSCs treated using AlPcS_4_Cl-AuNP and AlPcS_4_Cl-AuNP-Ab showed significant cell toxicity and cell death, compared to free AlPcS_4_Cl. The PDT effects were enhanced when using the NBC, showing significant lung CSC destruction to the point of eradication.

## 1. Introduction

Lung cancer is a life-threatening illness that accounts for about 20% of cancer-related deaths worldwide. The probability of relapse is a factor that contributes to the risk for cancer [1]. Chemotherapy and radiation are chemical and physical cancer treatments associated with surgery to enhance their effectiveness. These treatments, however, are not without their side effects, some of which include nausea, vomiting and diarrhea, hair loss and alopecia [2], general anesthesia and hospitalization for several days or weeks [3], mucositis, skin toxicity and xerostomia [4]. Cancer stem cells (CSCs) are a group of cells that have been reported as contributing to increased mortality in lung cancer. CSC tests have shown that conventional cancer treatments can be resisted [5]. Self-renewal, differentiation leading to phenotypic heterogeneity of tumor cells, metastases and recurrence of cancer is attributable to their stem-like characteristics [5,6]. Genetic expression of protein markers for drug efflux, recovery, and motility relates to the stem-like nature of CSCs [7]. The distinctive tendency of CSCs to repeat malignant development varies from the rest of the tumor, giving rise to both CSCs and cancer cells that are not stem-like [5]. Although little about CSC lung biology is currently recognized, different CSC markers have been identified and considered. These markers include ALDH1, CD133, Side Population (Hoechst-negative), CD44, CD87 and CD117. These factors are related to chemoresistance in various first-line cancer drugs [8]. The elimination of lung CSCs during therapeutic action is of the utmost importance, as this may stop the spread of CSCs, cancer recurrence and metastases. Research on CSCs due to resistance to conventional therapies and inability in complete eradication of cancer is critical for developing novel therapeutic strategies for a more effective reduction in the risk of tumor metastasis and cancer recurrence [9]. Due to the aforementioned concerns, the exploration of treatments that will have little to no side effects, target CSCs and are non-invasive has been initiated.

Photodynamic therapy (PDT) is based on the principle of light activation of a photosensitizing drug, causing tumor cell death [10]. PDT can perform selective cytotoxic action toward cancer cells through the administration of a photosensitizing drug with a wavelength equal to the absorbance range of the sensitizer caused by irradiation [11]. When Oxygen (O_2_) is available, PDT can cause tumor ischemia and starvation through indirect tumor ablation, seen by leaking of blood vessels, stopping blood flow and collapsing of blood vessels [12], and direct tumor cell destruction and local inflammatory reactions [13]. This is due to the formation of Reactive Oxygen Species (ROS) generated upon activation of the photosensitizer (PS) using light, inducing oxidative stress. These ROS cause cellular damage to the organelles and membranes of the cancerous tissue the PS is located in, leading to cell death via apoptosis or necrosis [14]. Up to date, PDT has been used palliatively for cancer, proving to be useful, with no PDT-related morbidity or mortality [15]. PDT cancer therapy has been widely accepted and used to treat a number of malignancies [16], as the procedure is safe to use, minimally invasive, has limited after-effects, is bio-interactive and compatible, can be repeated and is inexpensive [17,18]. Al(III) phthalocyanine chloride tetra sulfonic acid (AlPcS_4_Cl), a derivative of Photofrin, is a second-generation PS with a mixture of chlorine form and PS type phthalocyanine features [19]. Based on its emission spectrum of the Near-Infrared Region (NIR), AlPcS_4_Cl demonstrating deep tissue penetration has been shown to provide advantages over most PSs with high quantum generation, high water solubility, increased photo stability, poor photo bleaching and negligible dark cytotoxicity [12,20], all qualities of an optimal PS [19]. Despite the elevated achievement levels of PDT in the treatment of different cancers, there is still room for improvement by enhancing its drug delivery and making the PS target specific. This can be achieved by conjugating the PS to a delivery vehicle, such as gold nanoparticles (AuNPs), to enhance drug delivery and retention and an antibody providing a target-specific approach. These therapies are intended to specifically destroy CSCs, and not only reduce the tumor mass, but cancer as a whole, by enhancing its selectivity and efficacy [10].

Nanotechnology is a well-known field of study in which several biomedical applications and platforms have led to a research boom in diagnostic and therapeutic nanoscale agents [21]. Nanotechnology offers a platform for the modification and development of significant metal characteristics in the shape of nanoparticles (NPs) that encourage uses that are attributed to their distinctive physical, chemical and biological characteristics, such as surface resonance plasmon, polydispersity, stabilization and biocompatibility [22]. AuNPs incorporate these features, with respect to other biomedical nanodevices, and they provide a highly multifunctional system for disease identification and diagnosis. The complex synthesized architecture of AuNPs guarantees precise measurement of physicochemical and optical properties. The gold core is also harmless and is known to be biocompatible and non-toxic. For a specific application, the surface of AuNPs can be easily modified, and ligands, drugs and biocompatible coatings can be added [23,24]. AuNPs’ size provides enhanced permeability and retention effect (EPR) for many current large-molecular-weight medicines. AuNPs can be used to produce higher solubility and effective pharmacokinetics of drugs and imaging agents [25]. Passive targeting, active targeting or a combination of both approaches may be used to accomplish tumor-specific particle accumulation and drug delivery. The EPR effect, typical of many diseased sites, is used to passively kill cancer cells [26]. By combining AuNPs with multiple tumor targeting agents, such as antibodies (Abs), active targeting can be achieved. In relation to different targeting methods, the large surface-to-volume ratio of AuNPs allows for increased drug and/or prodrug load capacity, which can significantly reduce the minimum effective dosage relative to free-drug molecules [27]. Abs can efficiently and precisely target antigens for drug delivery, where Ab-conjugated PS is directed against a cancer, associated tumor (e.g., CSC) or vascular tumor antigen [28]. An advantage of Ab-directed phototherapy is that it provides cell-specific cytotoxicity of cancer cells, avoiding neighboring non-cancer tissues. In contrast, it is seen to be non-immunosuppressive and has no tendency for excessively divisive cells, which makes it less likely to cause treatment-induced resistance attributable to neoplastic cells that control alternate and circumventive mechanisms often seen in chemotherapy [29]. By using an Ab-nanobioconjugate (NBC), it can reduce both the PS clearance time and non-specific uptake into normal cells [30]. It has shown preservation of PS integrity and immunoreactivity and full stability, along with increased cytotoxicity and selective tumor targeting [31] making this form of PDT an ideal approach for cancer therapy, especially when considering the tumor’s heterogeneous population consisting of CSCs. In light of the above findings, PDT efficiency and specificity can be enhanced.

In this study, an NBC was constructed by using AlPcS_4_Cl, AuNPs and Abs; the compound was characterized in order to determine its stability and biocompatibility, and its photodynamic effect was explored in an in vitro setting. This was done in order to assess its potential as a cost-effective therapeutic method for the treatment of lung cancer, where the potential impacts of AlPcS_4_Cl cytotoxicity by AuNP pairing and antigenic targeting specifically for lung CSCs were established.

## 2. Results and Discussion

### 2.1. Characterization of Lung CSCs

#### Flow Cytometry

**Figure 1:**
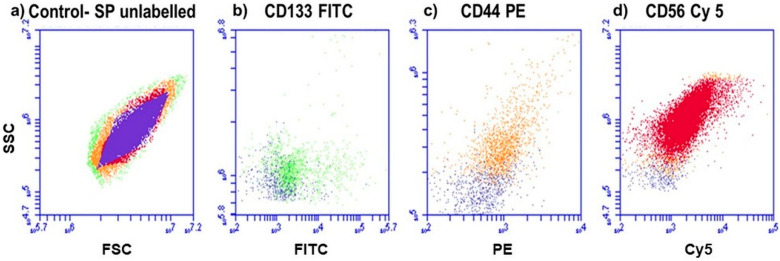
Fluorescence protein detection, using flow cytometry. (**a**) Control SP of cells that are unlabeled. (**b**) Lung CSCs positive for CD133 (FITC), (**c**) lung CSCs positive for CD44 (PE) and (**d**) lung CSCs positive for CD56 (Cy5). All positive samples are overlaid with the control, to distinguish between the color shifts.

Antigenic identification was evaluated by using flow cytometry, to determine whether the cells were of stem cell origin. Indirect Ab labeling permitted us to convey fluorescently whether the primary Ab on the cells was present or not. Fluorescence (FL) detection using flow cytometry showed that the indices CD133, CD44 and CD56 were detected on the cells, as shown in Figure 1.

CSCs isolated and identified within solid tumors, and cell lines have been shown to be capable of initiating tumor formation and differentiation in vitro [32]. Findings on how to recognize and isolate CSCs have been developed for the different variables involved in CSC studies, where the most common technique used to identify and isolate cell subpopulations (SPs) is the identification of protein markers or differentiation molecule clusters (CD markers) [33]. In this study, lung CSC markers were detected by using flow cytometric analysis, where antigenic expression of CD133, CD44 and CD56 was detected. These markers are prevalently expressed by stem cells representing the tissue of origin and were used to identify stemness within a cancer population [34].

### 2.2. Physicochemical Characterization of the NBC

**Figure 2:**
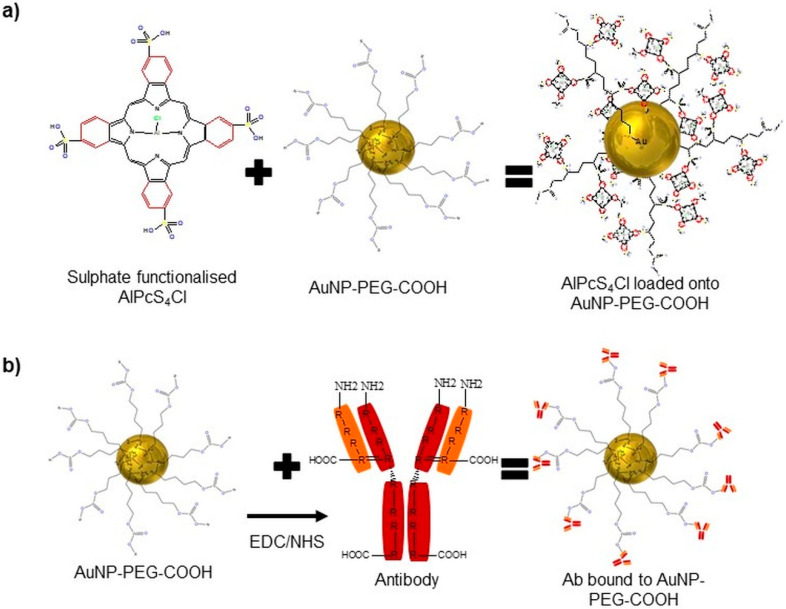
The NBC constructed using a PS (AlPcS_4_C), AuNPs (AuNP-PEG-COOH) and an Ab (Anti-CD133). (**a**) Loading of the sulphate functionalized PS onto the AuNPs via ligand exchange and adsorption. (**b**) Conjugating an Ab onto the AuNPs via EDC-NHS chemistry.

We constructed an NBC by loading the PS, AlPcS_4_Cl, onto AuNP-PEG-COOH via a centrifugation technique and attached an Ab (CD133) to the AuNP through EDC-NHS cross-coupling, allowing for the AuNPs to be coated with PS and Anti-CD133 (Figure 2).

Often, stabilizing polymers and biomolecules are altered to contain a -SH cluster to improve their conjugation on the surface of the Au. Polyethylene glycol (PEG)ylated AuNPs modified with linker molecules containing a thiol group at one end of the PEG terminal used to attach to the Au surface and a functional group of carboxylic acid (−COOH) at the other end was used to load AlPcS_4_Cl onto the AuNPs. This was done by charging the molecules, using a centrifugation technique that allows for ligand exchange between AuNPs and the PS sulfide groups and adsorption forming a disulphide bond between PEG and the PS [35]. Kang et al. used this form of conjugation to attach PEG to 30 nm AuNPs, which increased the stability of the particles in biological environments and allowed the subsequent conjugation of a nuclear-localizing peptide by free thiol groups located on the peptide at cysteine residues [36]. Other studies have also seen that, when loading AlPcS_4_ onto AuNPs, it increased the binding affinity and tumor selective uptake of the PS, along with enhanced singlet oxygen production caused by the EPR, and reduced the binding affinity of the PS to serum albumin [12]. This is due to the PEG stabilizing agent that surrounds the AuNP surface, creating a surface charge favoring steric repulsion; short-chain PEGs (PEG200) are seen to produce a negative surface charge of −28.2 mV, and with increased PEG chain lengths (PEG20000), the negative charge drops up to a minimum of −2 mV, diminishing the electrostatic repulsion [37]. In addition, through traditional EDC-NHS cross-coupling, the free functional group on the AuNPs-PEG-COOH gave the chance through electrostatic interactions to conjugate a range of biomolecules, such as DNA, Abs and polypeptides [38]. The -COOH that was functionalized into a primary amine allowed the Ab to bind to the AuNP via its c’ terminus, leaving the n’ terminus of the Anti-CD133 correctly orientated outward and unobstructed for active tumor biomarker recognition. CD133 has been postulated to identify CSC populations in numerous solid tumor types, including non-small-cell lung carcinomas and small-cell lung carcinomas [39]. Lung CSCs positive for CD133 had indicated indefinite tumor sphere formation [40], and it has been consistently reported that high levels of CD133 expression are associated with poor prognosis and aggressive cancer formation [41]. Some of the anticancer therapeutic approaches based on using overexpression of particular antigens, such as CD133, for targeted drug delivery, by selectively conjugating biomarkers such as Abs to the AuNP drug compound PDT results, can be further improved, as they can increase drug delivery and specificity [42].

#### 2.2.1. Quantification/Optical Properties/Stability—UV-Vis

**Figure 3:**
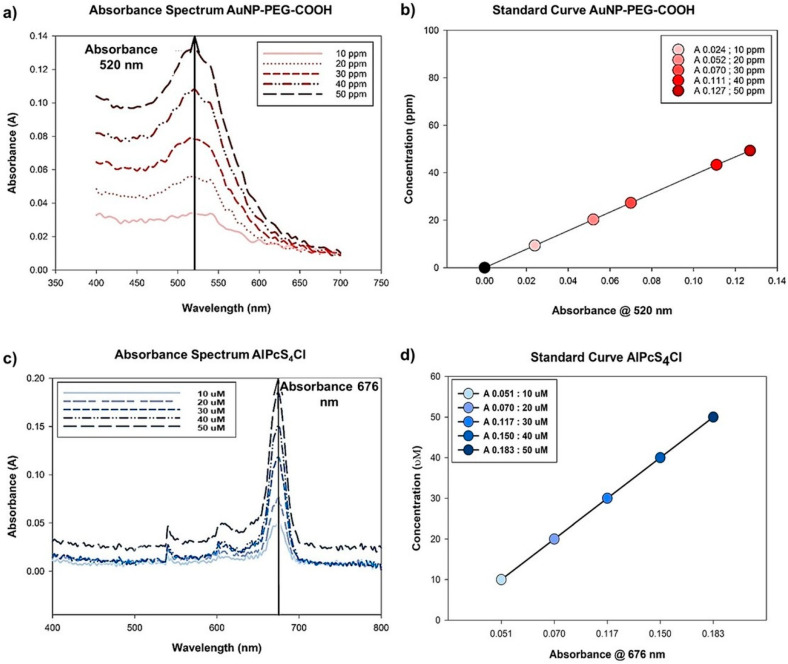
(**a**) Absorbance spectra of AuNP-PEG-COOH, showing an absorbance peak at 520 nm. (**b**) Standard curve of PEG-AuNP-COOH concentrations (10–50 ppm). (**c**) Absorbance spectra of AlPcS_4_Cl, showing an absorbance peak at 676 nm. (**d**) Standard curve of AlPcS_4_Cl concentrations (10–50 µM).

Absorbance spectra of AuNP-PEG-COOH and AlPcS_4_Cl are indicated in Figure 3a. In order to quantify the amount of PS loaded onto the AuNP-PEG-COOH, standard curves representing the molecules concentrations in relation to the absorbance measured were generated for AuNP-PEG-COOH and AlPcS_4_Cl (Figure 3b).

Ultraviolet-visible spectroscopy (UV-vis) of the single molecules demonstrated that AuNP-PEG-COOH has an absorption spectrum of 520 nm and the AlPcS_4_Cl an absorption spectrum of 676 nm. Both the AuNPs and the PS showed a linear distribution of concentration versus their dependent absorbances. The linear regression for each molecule was determined, and the regression coefficients calculated. The calculation for (**1**) AuNP-PEG-COOH and (**2**) AlPcS_4_Cl linear regressions are indicated in Table 1.

**Figure 4:**
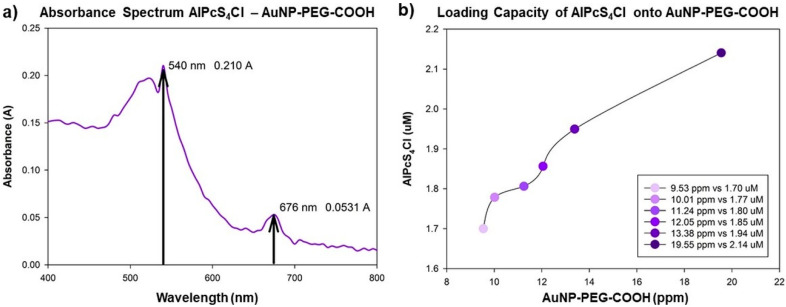
(**a**) Absorbance spectra of conjugated AuNP-AlPcS_4_Cl, with the particle’s respective absorbance peaks at 540 and 676 nm. (**b**) Distribution curve of the concentration of AlPcS_4_Cl (µM) relative to the concentration of AuNP (ppm).

After loading AlPcS_4_Cl onto the AuNP-PEG-COOH, the absorbance spectrum of the AlPcS_4_Cl-AuNP-PEG-COOH conjugate was measured; the conjugate demonstrates an absorption spectrum of 540 nm for the AuNPs and 676 nm for AlPcS_4_Cl (Figure 4a).

An absorbance shift was noted when comparing the wavelength of the single AuNP molecule with an absorbance peak at 520 nm initially to the AlPcS_4_Cl-AuNP-PEG-COOH conjugate having an absorbance of 540 nm, indicating a red shift. This red shift serves to confirm the adsorption of the PS molecule onto the AuNP [43]. After measuring the concentration of the PS conjugated to the various concentrations of AuNP used, as seen in Figure 4b, we could calculate the loading capacity of the AuNPs relative to the 20 µM AlPcS4Cl used for conjugation. Data analysis using the mean concentrations for the respective particles indicated that, to achieve ± 100% PS loading capacity, a ratio of 7 ppm: 1 µM of AuNP and PS can be used, respectively.

**Figure 5:**
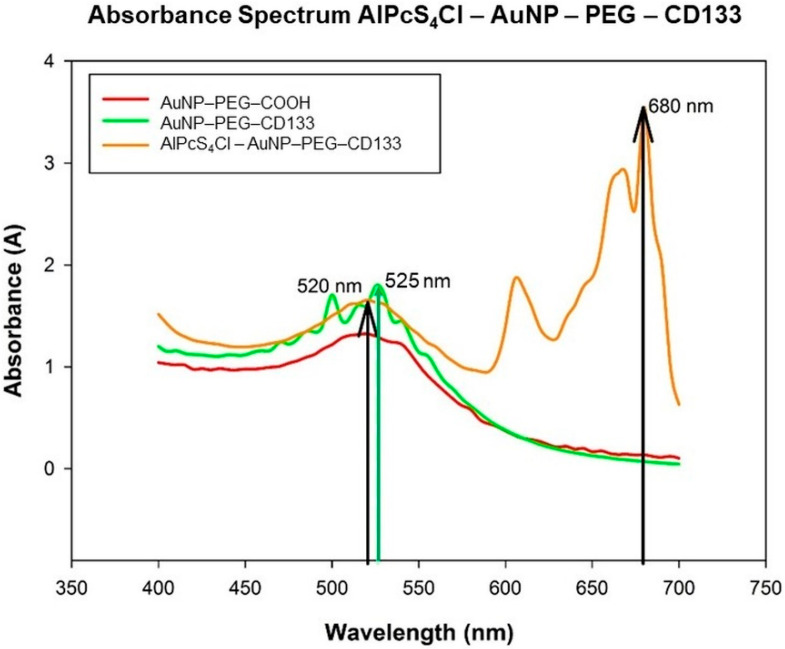
Absorbance spectrum of AuNP-PEG-COOH (red), AuNP-PEG-CD133 indicating a slight resonance peak shift at 525 nm due to the coupling of the Ab to the AuNP (green) and AlPcS_4_Cl-AuNP-PEG-CD133, with the particles respective absorbance peaks at 520 and 680 nm (orange).

Upon conjugation of the Ab (CD133) to the amine functionalized AuNP-PEG-NH, the conjugates’ absorption spectra were read, to ensure the efficient binding of CD133 to the surface of the particle and that the conjugate was stable. After that, the PS was loaded onto the AuNP-PEG-CD133 conjugate, and its absorbance spectra were measured (Figure 5).

A slight shift in resonance peak position of the nanoparticle before and after conjugation of the Anti-CD133 indicates that the Ab has been successfully conjugated to the surface. The AuNP-PEG-CD133 conjugate showed an absorption peak of 525 nm [44,45]. The PS was loaded onto the AuNP-PEG-CD133 for synthesizing a photodynamic NBC; the NBCs’ absorbance peak for the AuNP was seen to return to 520 nm, with the peak slightly broadening, indicating definitive bonding between all the chemical components; due to the increase in molecular size [44], the PS absorbance was established at 680 nm, which is indicative of the PS keeping its therapeutic window of photoactivation.

#### 2.2.2. Size and Surface Charge—Dynamic Light Scatter, Zeta Potential

The hydrodynamic size and zeta potential of the NBC, applying an indirect measuring method for Dynamic Light Scatter (DLS) and Laser Doppler Velocimetry (LDV) was used. The results are shown in Table 2.

The final NBC had an average size of 63.91 nm, so the composite can still be classified as a nano structure, as nanostructures are material configurations in the size range of 1–100 nm, where this physical parameter will allow for intracellular uptake and distribution [46]. The NBCs polydispersity index (PdI) was 0.497, suggesting that the NBC suspension was polydisperse with a moderate size distribution. The PdI, however, is still below the threshold of 0.7, for which measurements above the threshold are indicative of aggregation. In this case, the PdI can be ascribed to different primary sizes obtained upon conjugation of the Ab-CD133 and/or AlPcS_4_Cl to the AuNP [47]. The AuNPs zeta prospective values were −16.5 mV, the AlPcS_4_Cl-AuNP compound was read at −2.7 mV and the AlPcS_4_Cl-AuNP-CD133 zeta potential was −14.7 mV. The electrostatic charge of the particles indicates the stability of the sample in an aqueous environment. The negative charge of the AuNPs and conjugates are low due to relatively long PEG chains being functionalized to the AuNPs, shorter PEG chains would have indicated a negative charge which is higher [37]. Indicating the NBC overall steric repulsion to have an influence, the AlPcS_4_Cl-AuNP compound indicates a tendency for system destabilization through aggregation [48] where the composite half-life will diminish over time. The final NBC negative number indicates that the NBC is moderately stable with incipient instability [49], indicating that the steric repulsion of the NBC would be slightly diminished [37], which can be disadvantageous in vivo from the point of reticuloendothelial system (RES) recognition [50]. We therefore recommend ultra-sonication of the compound, along with confirming its stability before experimental use.

#### 2.2.3. Chemical Composition—FTIR

**Figure 6:**
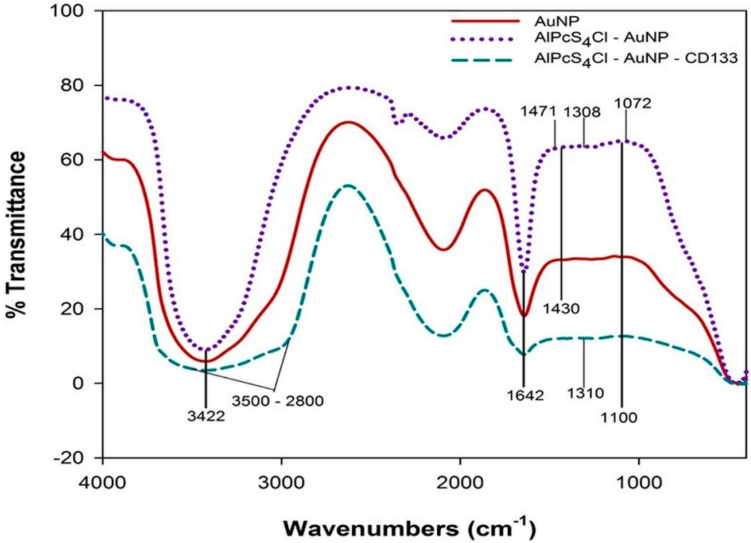
FTIR spectrum of AuNPs, AlPcS_4_Cl–AuNP conjugate and AlPcS_4_Cl–AuNP–CD133 conjugate.

Fourier-transform infrared spectroscopy (FTIR) was used for qualitative analysis of molecular ligand and absorption bond formations, as well as peptide bond identification [51]. Results are indicated in Figure 6. The AuNP-PEG-COOH indicated O-H stretching at 3422 cm^−1^ due to the compound being suspended in aqueous solution, C=C stretching at 1642 cm^−1^ was seen due to PEG functionalization, O-H bending was noted between 1413.9–1468.5 cm^−1^ due to carboxylic acid and C-O stretching at 1100 cm^−1^ due to the polyether structure was seen in PEG. AlPcS_4_Cl-AuNP-PEG–COOH also had O-H stretching at 3422 cm^−1^, C=C stretching at 1642 cm^−1^ was seen due to the PS structural components of its benzene rings, along with that of PEG found on the NP, C-H bending at 1471 cm^−1^ from the alkane methyl group from the PS structure was noted, O-H bending is seen due to carboxylic acid at 1430 cm^−1^, and S=O stretching at 1308.8 cm^−1^ and 1072 cm^−1^ was noted, from the sulphate functionalized PS. Furthermore, the PS – NP compound conjugated to primary Anti-CD133 also indicated S=O stretching at 1310.5 cm^−1^ from the sulphate functionalized PS being present, along with N-H stretching between 3500 and 2800 cm^−1^ due to the amine bonds present from the primary Ab conjugation.

FTIR shows that the AuNP functionalized with PEG-COOH was conjugated with AlPcS_4_Cl and CD133, which is confirmed by the PS – AuNP compound exhibiting all functional groups present from both compounds. In addition, the final NBC indicated that CD133 had conjugated to the AuNP-PEG-COOH compound, showing amide bonds present due to the terminus from the Ab binding to the n’ terminus on the AuNPs from the -COOH conversion.

### 2.3. Localization of the NBC in Lung CSCs—Fluorescence and Differential Interference Contrast Microscopy

**Figure 7:**
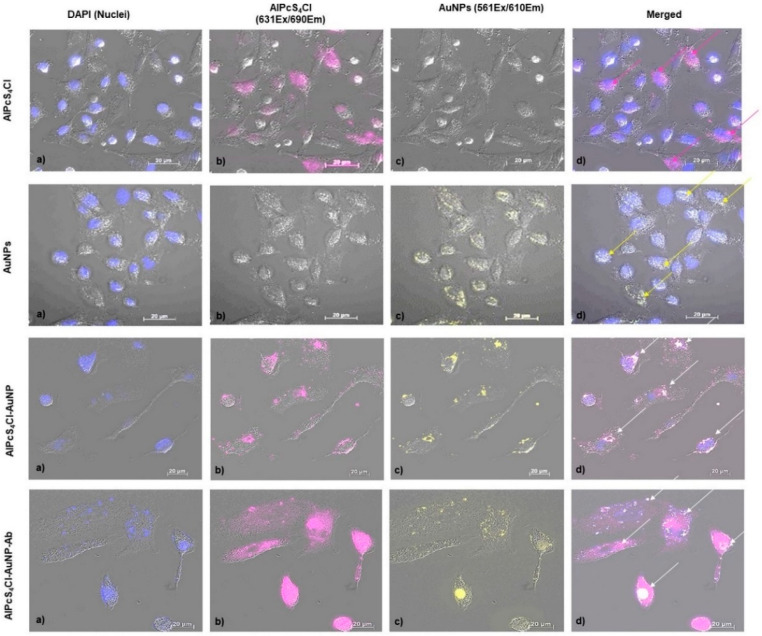
FL and DIC composite imaging of cellular localization of the NBC molecules and compounds in lung CSCs. (**a**) DIC imaging of lung CSCs, where cell nuclei are stained blue, using DAPI, (**b**) AlPcS_4_Cl auto fluorescence, (**c**) AuNPs auto fluorescence (**d**) and merged images of the reciprocal localization of the single NBC components in their different conjugation modes.

In order to establish the suitability of the NBC as a photodynamic drug delivery vehicle, the effectivity of delivering AlPcS_4_Cl into lung CSCs compared to free PS and its active uptake using Anti-CD133 was determined by analyzing cellular distribution of the molecules in lung CSCs. Localization was confirmed by using FL and differential interference contrast (DIC) microscopy, where the PS and AuNP auto fluorescence was exploited and detected using FL microscopy [52]. The cellular distribution of AlPcS_4_Cl and AuNPs as single molecules are indicated, as well as the compounds AlPcS_4_Cl-AuNP and AlPcS_4_Cl-AuNP-Ab (Figure 7**)**.

After analyzing the images, it showed that the NBC molecules had entered the cells. The AlPcS_4_Cl is localized in both the nucleus and cytoplasm. The AuNPs were noted to accumulate around the nucleus. The AlPcS_4_Cl-AuNP compound also showed peri-nuclear localization with the overlapping signal being cytoplasmatic due to the PS being conjugated to the AuNPs. However, an increase in AlPcS_4_Cl and AuNP concentration was seen when using the AlPcS_4_Cl-AuNP-Ab compound, indicated by an increase in fluorescence intensity. The accumulation of AlPcS_4_Cl, using the PS-AuNP conjugate, indicated similar localization patterns as the AuNPs. The PS-AuNP conjugate showed successful conjugation of the molecules, along with cell membrane permeabilization, where the PS concentrated around the nucleus, due to the small size of the AuNPs that passively entered the cells driving the delivery of the PS to subcellular locations [53]. The increased accumulation of AlPcS_4_Cl, by using the PS-AuNP-Ab conjugate, indicates active PS accumulation in the lung CSCs through Ab targeting [35], where the NBC has an increased affinity for the lung CSCs, allowing for improved uptake of the PS in the cytoplasm, perinuclear area and nucleus. The effects of PDT on lung cancer can be enhanced through both passive cancer cell targeting via the AuNPs and active targeting using Anti-CD133. The proposed mechanism for the uptake of the NBC is as follows. The AuNPs will allow for increased PS bioavailability, due to the NP size and PEG functionalization avoiding opsonization, by passive diffusion across the lipophilic cell membrane [54]. Furthermore, the Anti-CD133 conjugate efficiently guides the PS to the overexpressed CD133 markers at the tumor site, where the PS is internalized via endocytosis [55]. Upon Anti-CD133 ligand-receptor docking or receptor-mediated internalization, the process is followed by fusion with lysosomes [56]. The PS can then be released by photodynamic permeabilization of the lysosomal membrane re-localizing from the lysosomes to the cytoplasm in general and more specifically the nucleus [57]. This type of drug localization secures CSC death through damage of integral organelles involved in cell homeostasis [58].

### 2.4. Photodynamic Effects of the NBC on Isolated Lung CSCs

The photodynamic efficiency of the NBC was evaluated by using morphological examination, LDH Cytotoxicity, ATP Proliferation, Trypan Blue Viability and Annexin VPI Cell death analysis. The effects caused by the NBC were compared to that of the controls, as well as free AlPcS_4_Cl.

#### 2.4.1. Morphology

**Figure 8:**
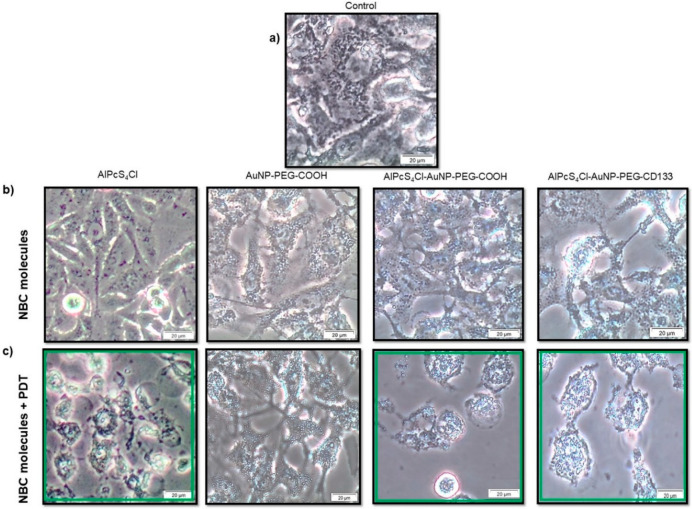
Morphology of lung CSCs 24 h post-treatment irradiation. (**a**) Untreated control cells, (**b**) CSCs receiving NBC molecules AlPcS_4_Cl, AuNP-PEG-COOH, AlPcS_4_Cl-AuNP-PEG-COOH and AlPcS_4_Cl-AuNP-PEG-CD133 without photo activation. (**c**) Cells receiving NBC molecules and irradiation.

Lung CSCs were morphologically characterized 24 h post-treatment with the NBC and irradiation, using inverted light microscopy (Figure 8). All treatment groups were compared to their respective controls not receiving photo activation, as well as the control sample not receiving any treatment.

Results indicated that cells receiving the NBC molecules without photo activation had no significant morphological changes when compared to the control cells receiving no treatment. Morphology shows no visible decrease in cell number, with no visible harm to cell membranes or signs of toxicity or cell death. This would suggest that the molecules alone had no dark toxicity, and this is characteristic of a good photosensitizing agent [59]. Cells that received AuNP-PEG-COOH with irradiation had no significant change in morphology when compared to their unirradiated control, and negligent vacuolization was noted due to internalization of the AuNPs. Lung CSCs that received AlPcS_4_Cl, AlPcS_4_Cl-AuNP-PEG-COOH and AlPcS_4_Cl-AuNP-PEG-CD133 with treatment irradiation showed morphological changes. The changes in morphological structure included cell shrinkage, chromatin condensation, cancer cell blebbing and cytoplasmic vacuolization, along with loss of cellular shape, detachment and free-floating cells that were observed. These changes are indicative of apoptotic cell death [60]. These findings would suggest that the AlPcS_4_Cl-AuNP-PEG-COOH conjugate passively enhanced PS uptake, and the final NBC (AlPcS_4_Cl-AuNP-PEG-CD133) actively enhanced PS-AuNP cellular uptake, leading to improved photodynamic action [61].

#### 2.4.2. Cytotoxicity/Proliferation/Viability/Cell Death

The PDT effects of the NBC was evaluated by using biochemical analyses and compared to their respective controls and that of free AlPcS_4_Cl that was previously established, as indicated in Table 3.

Biochemical analysis, including cytotoxicity, proliferation and viability, gave a clear indication of the cellular functions and how PDT affects the cells when using the NBC molecules and conjugates. Results are indicated in Table 4. Cytotoxic evaluation indicated no significant cellular toxicity when cells received the NBC molecules and conjugates without photo activation. Similarly, when evaluating cell proliferation and viability, cells showed optimum levels of cell function with no significant decrease. However, an increase in cytotoxicity was noted upon photo activation of the AlPcS_4_Cl-AuNP-PEG-COOH and AlPcS_4_Cl-AuNP-PEG-CD133 conjugates. Moreover, proliferation and viability had significantly decreased after irradiation using the photo-NBC compounds.

The negligent dark toxicity noted is in accordance with the literature stating that an ideal photosensitizing agent should have no toxic effect without photo activation [59]. It was also noted that, even after irradiation of the cells receiving AuNP-PEG-COOH, there was no significant change in cell function, and the slight changes seen could be attributed to photothermal effects induced by the irradiated AuNPs [62] which were insignificant. Studies have concluded that AuNP size, intracellular concentration and laser parameters highly influence the outcome [63], suggesting that the laser parameters and AuNP concentration used do not initiate cell inhibition via AuNP photothermal effects. Cytotoxicity was determined based on measuring the activity of cytoplasmic enzymes (LDH) released into the cell culture supernatant. The significant toxicity seen due to photoactivation of the photo-NBCs is indicative of plasma membrane damage due to cells undergoing apoptotic cell death [64]. Lung CSC proliferation measured by quantifying the amount of ATP available in the cells is indicative of the metabolic action of the cells [65]. Cellular ATP generated through aerobic glycolysis is produced by the mitochondria that produces energy in order to perform cellular processes [66]. The significant decrease in lung CSC ATP production, induced by the photodynamic action of the NBC, indicated that the PS had localized in subcellular organelles involved in cell homeostasis. The destruction caused to these organelles led to lung CSC inhibition, following apoptotic cell death [67]. Along with the significant increase in cytotoxicity and decrease in proliferation, the trypan blue dye exclusion assay confirmed cell inhibition and destruction seen by the decline in the percentage of viable cells post photo-NBC treatment. Cells that are undergoing or have undergone cell death had lost their membrane integrity allowing for the trypan blue dye to penetrate the cells [68]. The biochemical assays, as mentioned above, referring to cell membrane damage and cell count reduction, all corroborate the findings previously described in the morphological investigation.

Moreover, the photodynamic effects induced by the NBCs compared to those of free AlPcS_4_Cl showed a greater inhibitory effect on the lung CSCs, indicated by the significant increase in cytotoxicity and notable decrease in cell proliferation and viability. This would suggest that the use of nano carriers such as AuNPs, along with immunoconjugates, can significantly increase the potential of cell death when using AlPcS_4_Cl-PDT on lung cancer cells.

**Figure 9:**
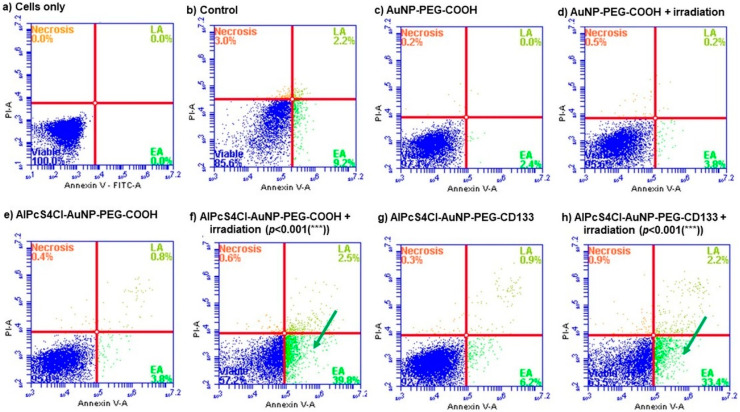
Annexin VPI cell death analysis of Lung CSCs 24 h post-irradiation. (**a**) Unstained cells were used to establish the cell population. (**b**) Cells receiving no treatment were used as the control. (**c**) Cells receiving AuNP-PEG-COOH alone and (**d**) AuNP-PEG-COOH with irradiation. (**e**) Cells receiving AlPcS_4_Cl-AuNP-PEG-COOH alone and (**f**) AlPcS_4_Cl-AuNP-PEG-COOH with irradiation (PDT). (**g**) Cells receiving AlPcS_4_Cl-AuNP-PEG-CD133 alone and (**h**) AlPcS_4_Cl-AuNP-PEG-CD133 with irradiation (PDT). Both (**f + h**) showed a significant increase in early apoptosis ((*p* < 0.001(***)).

The mechanism of cell death induced by the NBCs was confirmed by using the Annexin V PI cell death assay, which can differentiate between apoptotic and necrotic cell death [69]. Cell death analysis (Figure 9) indicated that, upon photo activation of the NBCs, apoptotic cell death was induced, shown by a significant increase in early apoptosis as compared to untreated cells and cells receiving NBC molecules without photo activation.

Photodynamic-induced cell death is caused by the formation of ROS through interaction of subcellular organelles, which relates to oxidative stress and subsequent cell damage by oxidizing and degrading cell components [70]. This type of oxidative stress can lead to cell death via apoptosis or necrosis [71]. Cell death via apoptosis was identified by Annexin V that bind to phosphatidylserine that is translocated to the outside of the plasma membrane [72]. Necrotic cells were identified by using propidium iodide (PI), a nucleic acid intercalator that penetrates porous cell membranes, allowing them to be attached to DNA [73]. Evidently, the use of photoactivated AlPcS_4_Cl- AuNP-PEG-COOH and AlPcS_4_Cl- AuNP-PEG-CD133 had led to the destruction of lung CSCs, inducing apoptosis. Moreover, an increase in early apoptosis was seen when comparing photoactivated NBCs to that of PDT alone, using only AlPcS_4_Cl. These findings suggest that, not only does the NBC photodynamic effect induce the preferred cell death mechanisms, but it also shows enhanced and effective lung CSC destruction.

## 3. Materials and Methods

A side population of epithelial lung carcinoma (A549) (ATCC^®^, CCL185™) CSCs, consisting of CD133^+^, CD44^+^ and CD56^+^ cells, was used in this study. The cells antigenic specificity was verified by characterization through Flow Cytometry, using indirect antigenic identification, where primary IgG Abs, CD133 Ab (3F10) (NovusBio, NBP2-3774), CD56 Monoclonal Anti-N Cam (Sigma, St. Louis, MO, USA, C 9672) and CD44 Ab (8E2F3) (NovusBio, Littleton, CO, USA, NBP1-47386) were used. An NBC was constructed, using (1) gold nanoparticles: AuNPs (Sigma-Aldrich, 765465) molecular weight 6.11 × 10^6^ Da, concentration 3000 ppm, that were 10 nm in diameter, PEG 5000 coated, carboxylic acid functionalized, with an OD of 50 dispersed in water and absorption wavelength of 530 nm; (2) purified CD133 Anti-Prom1 IgG (Abnova, Taipei, Taiwan, PAB12663); and (3) a PS (AlPcS_4_Cl) (Frontier Scientific, Logan, UT, USA, AlPcS-834) with the formula C32H16AlClN8O12S4 and formula weight 895.21, TLC > 95% with an absorbance wavelength of 676 nm. Localization and uptake of the selected NBC molecules in lung CSCs were determined by using FL and DIC microscopy and spectrophotometry. The molecules were conjugated by using a centrifugation technique and EDC-NHS chemistry. The newly synthesized NBC was characterized by using FL and DIC inverted microscopy (Inverted Fluorescence Microscope, Zeiss, Axio Observer Z1, Carl Zeiss (Pty) Limited, Gauteng, South Africa), ATR-FTIR spectroscopy (Nicolet 380 FT-IR Spectrometer, Thermo Fisher Scientific, Gauteng, South Africa), UV-Vis spectroscopy (Genova Nano Micro-volume Life Science Spectrophotometer, Jenway^®^, 737501, Lasec SA (Pty) Ltd., Cape Town, South Africa) and DLS and Zeta-potential/LDV (Zetasizer Nano ZS, ZEN 3600, Malvern Panalytical (Pty) Ltd., Gauteng, South Africa). The effects of TPDT 24 h post laser irradiation, using the newly synthesized NBC, were determined by biochemical assays, including morphology, LDH cytotoxicity, ATP proliferation, Trypan blue viability and Annexin VPI cell death. All solvents and reagents were reagent-grade and were used as received.

### 3.1. Cell Culture

Cells were cultured in Rosewell Park Memorial Institute 1640 medium (RPMI-1640) (Sigma-Aldrich, R8758) supplemented with 10% fetal bovine serum (FBS) (Sigma-Aldrich, S0615) and 0.5% penicillin/streptomycin (Sigma-Aldrich, P4333) and 0.5% amphotericin B (Sigma-Aldrich, A2942). Cell lines enriched for the respective markers were maintained at a passage between 4 and 8 for all experimental purposes. All cultures were maintained and incubated at 37 °C in 5% CO_2_ and 85% humidity.

### 3.2. Characterization of the Subpopulation of Lung CSCs

#### Flow Cytometry

To confirm whether the SP of cells were of CSC origin, cells were characterized, using a flow cytometric assay to determine antigenic positivity. The secondary Ab conjugation technique was used where the subpopulation was labeled with primary mouse anti-human Ab CD133, CD44 and CD56 and fluorescently marked with secondary FITC Goat anti-Mouse (NovusBio, NB720-F-1mg), PE Goat anti-Mouse (NovusBio, NB7594) and Cy5 Goat anti-Mouse (NovusBio, NB7602) antibodies, respectively. Antigenic detection was determined by using the BD Accuri^TM^ C6 Flow Cytometer (BD Biosciences, BD ACCURI C6 PLUS, BD Becton Dickinson, Gauteng, South Africa), and results were depicted qualitatively.

### 3.3. Synthesizing the Nano Bio-Composite

#### 3.3.1. Conjugation of AlPcS_4_Cl to AuNP-PEG-COOH

A centrifugation technique allowing for ligand exchange and adsorption of the PS sulphate groups to the free thiol groups on the Au and PEG chains was used [51]. AlPcS_4_Cl was added at a concentration of 20 µM to various concentrations of AuNP (10–50 ppm) in 2 mL Eppendorf^®^ LoBind micro centrifuge tubes (Sigma, Z666556). The mixtures were stirred at high speed, overnight, at room temperature (RT), with negligible light exposure, using a Multifunction Vortex Mixer Set with a Microtube Platform Head (VM-10) (DAIHAN-brand^®^, N05042000008187, DAIHAN Scientific Daegu Gyeongbuk Branch Co., Ltd., South Korea), allowing for gentle mixing of both compounds with a constant circular, oscillating movement.

To remove the free AlPcS_4_Cl from the charged complex, the mixture was purified by repeat centrifugation, at 15,200 rpm for 1 h (Heraeus™ Fresco™ 17 Micro centrifuge, Thermo Scientific™, 75002402, Thermo Fisher Scientific, Gauteng, South Africa). Then the supernatant was removed and the product solubilized and centrifuged until unbound PS was removed. The conjugate was then suspended in 1× Phosphate buffered saline (PBS) (Sigma-Aldrich, P3813) and analyzed, using a Spectrophotometer, in order to establish the ratio of PS loaded. After, a batch of AuNP-PS conjugate was made to use for further experimental purposes. The conjugate was solubilized in ddH_2_O and stored at 4 °C in the dark.

#### 3.3.2. Conjugation of Abs to AuNP-PEG-COOH

A general two-step covalent coupling protocol for conjugation of proteins to carboxylic acid functionalized AuNPs, using EDC and Sulfo-NHS, was used. In order to convert the carboxyl group into a primary amine-reactive N-hydroxy succinimide ester (NHS), the following adapted protocol was used [74,75]: EDC/NHS solution ((1-Ethyl-3-[3-dimethylaminopropyl]carbodiimide hydrochloride (EDC) (Sigma-Aldrich, 03449) N-Hydroxysulfosuccinimide sodium salt (Sulfo-NHS) (Sigma-Aldrich, 56485)) dissolved in MES (10 mM 2-N-morpholino ethane sulfonic acid (MES) (Sigma-Aldrich, M3671), pH 5.5) was added to 0.5 mg/mL PEG-AuNP-COOH into a micro centrifuge tube with a final EDC/NHS concentration of 50 mg/mL EDC and 50 mg/mL NHS and incubated for 30 min at RT in the dark; 500 µL PBS was added and vortexed as a washing step; the mixture was then centrifuged at 15,200 rpm for 1 h at 4 °C, and the supernatant was removed after conjugation of the Abs commenced, immediately.

Ab solution of 5 µg/mL (from stock concentration of 15 µg/mL) was transferred to the activated AuNPs and mixed thoroughly to re-solubilize the AuNPs and incubated at RT for 2 h; 500 µL of 1× PBS was added and the mixture washed and centrifuged at 15,200 rpm for 1 h, and the supernatant was removed; 500 µL of blocking buffer (1× PBS, 1% (*w*/*v*) Bovine Serum Albumin (Sigma-Aldrich, A2153)) was added and mixed for 30 min, in order to reduce the effects of nonspecific binding; the solution was centrifuged at 15,200 rpm for 1 h, and the supernatant was removed; the volume was adjusted to 500 µL, using storage buffer (1× PBS); 500 µL PS was then loaded onto the AuNP-PEG-Ab, resulting in a final concentration of 500 µM AlPcS_4_Cl, as explained above, without removal of free PS, and the conjugate was measured spectrophotometrically. The newly synthesized NBC was stored at 4 °C, in the dark, for experimental use.

### 3.4. Physicochemical Characterization of the Newly Synthesized NBC

#### 3.4.1. Optical Properties/Quantification/Stability—UV-Vis

For spectroscopic analysis stock solutions consisting of 1 mM AlPcS_4_Cl, 3000 ppm AuNP was aliquoted into single concentrations of 10–50 µM PS and 10–50 ppm AuNP, in order to produce a standard curve, where UV-vis absorbance was measured in the spectral range of 400–800 nm for the PS and NP. The conjugated molecules, consisting of AuNP-PS and AuNP-PS-Ab, were also analyzed spectrophotometrically. From the standard curves generated, we could determine the concentration of AuNP loaded with AlPcS_4_Cl after conjugation and establish a ratio for the PS-to-NP loading capacity. From these values, stock solutions consisting of AuNP-PS conjugate and PS-AuNP-Ab conjugates with a PS concentration of 500 µM and AuNP of 500 ppm were made and stored at 4 °C. All samples for Bio and Physiochemical characterization were prepared as single molecules and conjugates, as explained above.

#### 3.4.2. Size and Surface Charge—DLS, Zeta Potential

Zeta potential is an important tool for understanding the state of the nanoparticle surface and predicting the long-term stability of the nanoparticle. DLS and LDV, for characterization of size and zeta potential of the NPs in solution, were performed by using the Zetasizer Nano ZS. Samples were prepared as single-molecule suspensions for AuNPs, at a concentration of 20 ppm, and conjugated suspensions of PS-AuNP and PS-AuNP-Ab with PS and AuNP concentrations of 50 µM and 50 ppm, respectively. All samples were dispersed in water (Dispersant RI: 1.330, Viscosity (cP): 0.8872). The solutions were vortexed to provide a homogeneous suspension, where 1 mL of each sample was transferred to a disposable sizing cuvette for DLS measurements, and zeta Dipcell was used for LDV measurements that were read at 25 °C.

#### 3.4.3. Chemical Composition—FTIR

In order to obtain an infrared spectrum of absorption, emission, photoconductivity or Raman scattering, FTIR was used. The NBC molecules’ connections and functional groups were analyzed by the FTIR spectrometer. FTIR readings were performed by using a spectrometer fitted with an ATR crystal testing device (UATR Accessory, Ge (1 Reflection), L1250054, PerkinElmer, Gauteng, South Africa) that allows samples to be immediately examined in liquid state. Measurements were performed in the 400–4000 cm^−1^ spectral range. In order to improve the signal-to-noise percentage, each spectrum was an average of 64 tests.

### 3.5. Localization of the NBC Molecules in Isolated Lung CSCs—Fluorescence and Differential Interference Contrast Microscopy

To determine the suitability of the NBC molecules and the newly synthesized compound (AuNPs, AlPcS_4_Cl and CD133) for TPDT, localization and uptake of the NBC molecules into isolated lung CSCs was confirmed by using FL and DIC microscopy. Cells were grown on Microscope coverslips (MARIENFELD, 0895202, Lasec SA (Pty) Ltd., Cape Town, South Africa) in culture dishes (Corning^®^, Sigma, CLS430588) in complete media, at a seeding density of 5 × 10^5^ cells, until the cells attached. Once the cells were attached, they received AlPcS_4_Cl at a concentration of 20 µM, which was used as the treatment concentration for all experimental purposes throughout the study and 20 ppm AuNPs. The cells were incubated for at least 4 h, allowing the PS and AuNPs to penetrate the cells. Cell nuclei were stained using 4′,6-diamidino-2-phenylindole, dihydrochloride (DAPI) (Invitrogen™, D1306) 358Ex/461Em. FL and DIC microscopy were performed, using an Inverted Fluorescence Microscope. Intracellular detection of the PS was measured by auto fluorescence of the AlPcS_4_Cl, using a 631Ex/690Em filter. To determine the localization of the AuNPs, the auto fluorescence of the particles was measured, using a 561Ex/610Em filter. The images were compiled by using a Java image-processing program, ImageJ, developed at the National Institutes of Health and the Laboratory for Optical and Computational Instrumentation, License: Public Domain, BSD-2.

### 3.6. Photodynamic Effects of the NBC on Isolated Lung CSCs

In order to determine the photodynamic efficiency of the newly synthesized NBC used as a photodynamic compound and drug delivery vehicle on isolated lung CSCs, we evaluated the PDT effects by using biochemical analysis. A predetermined concentration and laser fluence for the PS was used, which determined the concentration of AuNP the cells would receive. Laser parameters and NBC molecule concentrations are seen in Table 5. The ratio of PS loaded onto the AuNPs were used as 1 µM PS:1 ppm AuNP, in order to preserve cells for downstream applications. This is due to the free AlPcS_4_Cl used at a concentration of 20 µM on lung CSCs, indicating [IC50], and that, should the AuNP and Ab increase the photodynamic action of AlPcS_4_Cl, biochemical assays could still be performed.

The cells were irradiated with a 673.2 nm Diode Laser (National Laser Centre of South Africa, SN 070900108, Gauteng, South Africa), with a 1000 mA LaserSource (4210 LaserSource, Arroyo Instruments, LLC, CA, United States). The laser output power (mW) was measured by using a FieldMate Laser Power Meter (Coherent^®^, FieldMate, 1098297, Alp Applied Laser Power, Western Cape, South Africa) and a High-Sensitivity Thermopile Sensor PM3 (Coherent^®^, PM3,1098336, Alp Applied Laser Power, Western Cape, South Africa) used to calculate the laser exposure time based on the fluence. The cells were cultured in a 3.4 cm Petri dish, at a concentration of 5 × 10^5^ cells, and allowed to attach for 4 h; then the PS was added. The magnitude of the laser spot filled the entire cell monolayer. The light was produced from a fiber optic 8 cm above the cells. The Petri dish lid was removed, to avoid light scattering. All studies were conducted in the dark, at RT, to avoid factors of nuisance. The calculation for irradiation time was as follows:

(1) $$mW/cm^{2\;}=\frac{mW\times 4}{\pi \times 3.4^{2}}$$

(2) $$W/cm^{2}=\frac{mW/cm^{2\;}}{1000}$$

(3) $$Time\;\left(s\right)=\frac{J/cm^{2}}{W/cm^{2}}$$

Cell cultures were split into four fields of analysis. Group 1 was the control and received no irradiation or PS; group 2 (a) contained AuNP but no irradiation, and group 2 (b) received AuNP and irradiation; group 3 (a) contained AuNP and PS, and group 3 (b) received AuNP and PDT; group 4 (a) contained AuNP, PS and Ab; and group 4 (b) received TPDT, using AuNP, PS, Ab and irradiation. All samples were incubated for 24 h after irradiation treatment, followed by biochemical analysis.

#### 3.6.1. Morphology

Differences in morphology were noted and evaluated 24 h after therapy, using an Inverted Microscope (OLYMPUS, CKX41, Wirsam Scientific & Precision Eq. Ltd., Gauteng, South Africa), and recorded by using a microscope-connected digital camera (OLYMPUS, SC30, Wirsam Scientific & Precision Eq. Ltd., Gauteng, South Africa), which utilizes the getIT software that shows pictures visually, along with their size scale.

#### 3.6.2. Cytotoxicity

The CytoTox 96^®^ non-radioactive cytotoxicity assay (Promega, G1780) was used to determine cellular toxicity. Where the outcomes were colorimetrically recorded, using the 490 nm reading of the VICTOR3 Multilabel Plate Counter (PerkinElmer, 1420-014, PerkinElmer Life and Analytical Sciences, CT, United States).

#### 3.6.3. Proliferation

Cell proliferation was determined by using the CellTiter-Glo^®^ 2.0 (Promega, G9241) ATP luminescence assay. The luminescent signal was read and quantified, using a Multilabel Plate Counter, depicting the luminescent signal as relative light units (RLUs).

#### 3.6.4. Viability

A color-exclusion experiment with Trypan Blue Stain (0.4%) (Invitrogen™, T10282) was used to determine cell viability. The Automated Cell Counter (Countess™ II Automated Cell Counter, AMQAX1000, Thermo Fisher Scientific, Gauteng, South Africa), which visually depicts the cells and calculates the percentage of viable cells per mL, was used to count viable cells, excluding dye and dead cells retaining the dye.

#### 3.6.5. Cell Death

An FITC Annex V Apoptosis Detection Kit I (BD Pharmingen™, 556547, BD Becton Dickinson, Gauteng, South Africa) was used to measure cell death. The assay utilizes a fluid cytometer to read and quantify FL signals from necrotic or apoptotic cells.

### 3.7. Statistics

All investigations were conducted in three repeats. Data analysis was carried by out using version 13 of the Sigma plot. Error bars are the median (SEM) bar (*n* = 3). The collected data were assessed statistically by the Student’s paired t-test, and the significance was defined as *p* < 0.05(*), *p* < 0.01(**) or *p* < 0.001(***). All mass spectroscopy tests were carried out by using a background check deducted from the relevant data extracted.

## 4. Conclusions

The various molecular characterization assays confirmed that AlPcS_4_Cl PS drug was successfully conjugated onto the surface of AuNPs via ligand absorption and exchange methods. Additionally, these same assays noted effective c’ terminus amide bonding of Anti-CD133 Abs onto the surface of the amine functionalized AuNPs, leaving the n’ terminus of the Anti-Ab correctly orientated outward and unobstructed for active tumor biomarker recognition. Furthermore, DLS and Zeta Potential results in relation to the final PS drug conjugate noted that the NP drug system was of nano-size, polydisperse with a moderate size distribution and moderate stability, and so it could be considered as an effective PDT PS drug carrier. Moreover, the subcellular localization results reported that the NBC was efficient at improving AlPcS_4_Cl intracellular accumulation within the cytoplasm and nuclei of cells, due to its Anti-CD133 Ab biomarker targeting affinity for lung CSCs.

The findings from this study suggest that the conjugation of AlPcS_4_Cl to AuNPs and Anti-CD133 actively and specifically enhanced AlPcS_4_Cl PS drug uptake in lung CSCs. This mode of delivery greatly improved the transport of the drug to the cells, compared to AlPcS_4_Cl alone. This is proven by the significant increase in cytotoxicity and apoptosis seen using the NBC compared to free AlPcS_4_Cl and their controls, along with the dramatic decline in lung CSC proliferation and viability. These findings would suggest that AlPcS_4_Cl PS drug delivery within in vitro cultured lung CSCs can be improved by using active AuNP-Ab targeting, and can enhance the overall outcomes of PDT treatment on lung cancer cells.

Future recommendations for the physical characterization of the NBC can include complimentary thermal gravimetric analysis (TGA) and transmission electron microscopy (TEM) studies to further validate the claims of the NBC in relation to the nanoparticles’ thermal stability and structural composition respectively [76]. Physicochemical analysis of the molecular variation of NBCs due to protein aggregation should also be investigated. This would make it easier to assess the variations if the sample structure that may be induced during synthesis. Protein aggregation can be measured by evaluating the fluorescence spectra of the NBC [77]. Moreover, the mechanisms involved in photoactivated NBC cell death leading to free radical apoptosis of CSCs remain unknown, as cell death may be caused by distinct, but overlapping, signaling pathways that respond to different stimuli [78]. Cell death pathways and signaling mechanisms can be studied by numerous metabolic studies, including RT-PCR caspase signaling identification, Cytochrome C and ROS identification and DNA destruction [79]. The aforementioned recommendations will aim to address any limitations observed in this report. Furthermore, the efficacy and selectivity of PDT as a lung cancer treatment can further be explored in vivo by utilizing the same AuNP-Ab construct as a molecular target and be further developed for clinical trial purposes.

## Figures and Tables

**Table 1:** Linear regression of the calibration curves for (**1**) AuNP-PEG-COOH and (**2**) AlPcS_4_Cl.

(1) ŷ = 0.00265*X* − 0.0027	(2) ŷ = 0.00344*X* + 0.011
Sum of *X* = 150 Sum of *Y* = 0.384 Mean *X* = 30 Mean *Y* = 0.0768 Sum of squares (*SSX*) = (X − Mx)2 = 1000 Sum of products (**SP**) = (X − Mx)(Y − My) = 2.65	Sum of *X* = 150 Sum of *Y* = 0.571 Mean *X* = 30 Mean *Y* = 0.1142 Sum of squares (*SS_X_*) = (X − M_x_)^2^ = 1000 Sum of products (*SP*) = (X − M_x_)(Y − M_y_) = 3.44
Regression Equation = ŷ = *bX + a* *b* = *SP/SSX* = 2.65/1000 = 0.00265 *a* = MY − *b*M_X_ = 0.08 − (0 × 30) = −0.0027 ŷ = 0.00265*X* − 0.0027	Regression Equation = ŷ = *bX + a* *b* = *SP/SSX* = 3.44/1000 = 0.00344 *a* = MY − *b*M_X_ = 0.11 − (0 × 30) = 0.011 ŷ = 0.00344*X* + 0.011

**Table 2:** DLS and LDV measurements of the single AuNP suspension, AlPcS_4_Cl-AuNP and AlPcS_4_Cl-AuNP- CD133.

Sample	Z-Average (d.nm)	PdI	Zeta Potential (mV)
AuNP	49.48	0.357	−16.5
AlPcS_4_Cl-AuNP	61.99	0.477	−2.7
AlPcS_4_Cl-AuNP-CD133	63.91	0.497	−14.7

**Table 3:** Effects of free AlPcS_4_Cl on lung CSCs using 20 µM PS and irradiation of 10 J/cm^2^.

Biochemical Assay	Cytotoxicity	Proliferation	Viability	Cell Death Mode		
				Early Apoptosis	Late Apoptosis	Necrosis
Percentage (%)	43.33 ± 1.1	16.66 ± 4.7	44 ± 7	21 ± 4.7	2.6 ± 0.2	9 ± 3.3

**Table 4:** Biochemical evaluation of the NBC photodynamic effects in lung CSCs 24 h post-irradiation. A statistically significant increase in cytotoxicity and decrease in proliferation and viability was seen post-irradiation, using AlPcS_4_Cl-AuNP-PEG-COOH and AlPcS_4_Cl-AuNP-PEG-CD133 (*p* < 0.001(***)).

	Biochemical Assay		
Experimental Group	Cytotoxicity (%)	Proliferation (%)	Viability (%)
Control	15.3 ± 9.06	86.54 ± 6.5	76 ± 1.73
AuNP-PEG-COOH	32.47 ± 9.69	84.77 ± 6.59	80.66 ± 4.66
AuNP-PEG-COOH + irradiation	38.26 ± 16.09	93.51 ± 2.27	81.66 ± 5.23
AlPcS_4_Cl- AuNP-PEG-COOH	35.96 ± 6.66	84.35 ± 10.6	86.66 ± 3.75
AlPcS_4_Cl- AuNP-PEG-COOH + irradiation	(***) 97.07 ± 2.74	(***) 7.72 ± 8.36	(***) 30.33 ± 2.33
AlPcS_4_Cl- AuNP-PEG-CD133	27.73 ± 6.38	100 ± 6.74	72.33 ± 2.4
AlPcS_4_Cl- AuNP-PEG-CD133 + irradiation	(***) 93.4 ± 6.39	(***) 10.59 ± 13.9	(***) 15.33 ± 1.76

**Table 5:** NBC and laser parameters using the 673.2 nm diode laser.

PARAMETERS	
Laser type	Semiconductor (Diode)
Wavelength (nm)	673.2
Wave emission	Continuous
Fluence (J/cm^2^)	10
Nano-bio-composite (NBC)	AuNP-PEG-AlPcS_4_Cl-Ab
PS Concentration (µM)	20
AuNP Concentration (ppm)	20

## Abbreviations

CSCs	Cancer stem cells
PDT	Photodynamic therapy
PS	Photosensitizer
AlPcS_4_Cl	Al (III) phthalocyanine chloride tetra sulfonic acid
NIR	Near-Infrared
AuNPs	gold nanoparticles
EPR	enhanced permeability and retention
Abs	antibodies
TPDT	targeted PDT
NBC	nanobioconjugate
FL	fluorescent
DIC	differential interference contrast
ATR-FTIR	Attenuated Total Reflection Fourier Transform Infrared spectroscopy
UV-Vis	ultraviolet-visible spectroscopy
DLS	Dynamic Light Scatter
LDV	Laser Doppler Velocimetry
FBS	fetal bovine serum
SP	sub population
RT	room temperature
PBS	phosphate buffered saline
NHS	N-hydroxy succinimide ester
EDC	1-Ethyl-3-[3-dimethylaminopropyl]carbodiimide hydrochloride
PdI	polydispersity index
